# Tumor suppressor miR-317 and lncRNA Peony are expressed from a polycistronic non-coding RNA locus that regulates germline differentiation and testis morphology

**DOI:** 10.1101/2024.10.10.617551

**Published:** 2024-10-10

**Authors:** Travis D. Carney, Halyna R. Shcherbata

**Affiliations:** 1 Institute of Cell Biochemistry, Hannover Medical School, Carl-Neuberg-Strasse 1, 30625, Hannover, Germany; 2 Mount Desert Island Biological Laboratory, Bar Harbor, ME 04609, USA

**Keywords:** miRNA, lncRNA, gene expression, germline tumor, Notch signaling, tumor suppressor, Drosophila testes

## Abstract

This research focuses on investigating the impact of non-coding RNAs on stem cell biology and differentiation processes. We found that *miR-317* plays a role in germline stem cell progeny differentiation. *miR-317* and its neighbor, the lncRNA *Peony*, originate and are co-expressed from a singular polycistronic non-coding RNA locus. Alternative polyadenylation is implicated in regulation of their differential expression. While the increased expression of the lncRNA *Peony* results in the disruption of the muscle sheath covering the testis, the absence of *miR-317* leads to the emergence of germline tumors in young flies. The deficiency of *miR-317* increases Notch signaling activity in the somatic cyst cells, which drives germline tumorigenesis. Germline tumors also arise from upregulation of several predicted targets of *miR-317*, among which are regulators of the Notch pathway. This implicates *miR-317* as a novel tumor suppressor that modulates Notch signaling strength.

## Introduction

Three decades of research have revealed the involvement of microRNAs (miRNAs) in many aspects of stem cell biology and in processes of differentiation. MiRNAs have been shown to modulate cell division in human embryonic stem cells by targeting key cell cycle checkpoint components (Qi et al. 2009). In Drosophila, miRNAs are required for cell cycle progression as well as maintenance of ovarian germline stem cells (GSCs) (Hatfield et al. 2005; Shcherbata et al. 2007). In addition to roles in stem cell function and homeostasis, we have previously shown that miRNAs affect aspects of cell fate determination in multiple cell types in Drosophila (Kucherenko et al. 2012; Yatsenko and Shcherbata 2014). These studies demonstrate the importance of miRNAs in the crucial processes of stem cell function and cellular specification and differentiation.

**Figure 1.**
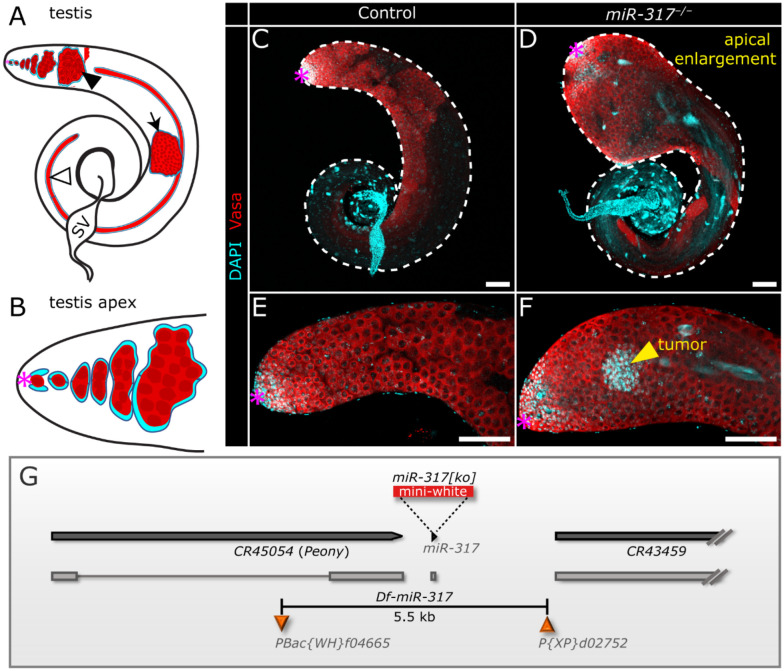
*miR-317[ko]* causes testis phenotypes in adult males. **A** Schematic of one adult testis. Shown are stem cell niche (hub; magenta asterisk); somatic cyst cells surrounding germline cysts (cyan color); 16-cell spermatocyte cyst (solid arrowhead); 64-cell post-meiotic cyst (black arrow); elongated spermatid-stage cyst (open arrowhead); and seminal vesicle (SV). **B** Testis apex. Hub (magenta asterisk); germline cells (red) surrounded by somatic cyst cells (cyan). Germline cells divide to form 16-cell cysts. **C** Control testis is a coiled, closed-ended tube (outlined with white dashed line). **D** *miR-317*^−/−^ testis exhibiting apical enlargement phenotype. **E** Control testis apex. **F** *miR-317*^−/−^ apex containing a tumor (yellow arrowhead), identified by strong expression of Vasa (red) as well as strong DAPI staining (cyan) distal from the apex. **G** Genomic context of *miR-317*, which is closely apposed to the lncRNA gene *CR45054 (Peony). miR-317[ko]* is a knock-in mutant in which the pre-miRNA is replaced by a *mini-white* cassette. *Df-miR-317* is a 5.5-kb deletion of *miR-317* and the large second exon of *Peony*. Scale bars: 100 μm.

The Drosophila testis has proven to be an invaluable model for the study of stem cell biology, intercellular signaling, and differentiation. The testis is a coiled tube, closed at the apical (anterior) end, in which the processes of spermatogenesis take place (Figure 1A). At the testis apex, a cluster of specialized somatic cells called the hub forms a stem cell niche capable of supporting and maintaining two populations of stem cells: germline stem cells (GSCs) and somatic cyst stem cells (CySCs) (de Cuevas and Matunis 2011) (Figure 1B). Both are maintained through direct contact with the hub and through multiple cell-signaling pathways. GSCs and CySCs divide asymmetrically such that one daughter cell retains both niche contact and stemness, while the other daughter, no longer proximal to the self-renewal-promoting signals from the niche, begins a differentiation program. The differentiating daughter of the GSC, a gonialblast, becomes encapsulated by two somatic daughter cells, cyst cells, which themselves differentiate in concert with the germline cells and maintain this association throughout the rest of spermatogenesis. Cyst cells serve as a differentiation niche, supporting spermatogenesis processes and protecting the germline cells from exogenous signals (Decotto and Spradling 2005; Zoller and Schulz 2012; Papagiannouli 2014). The gonialblast undergoes four rounds of transit-amplifying mitotic divisions to form a 16-cell cluster of cells termed spermatogonia (Greenspan et al. 2015). This cell cluster and the two encapsulating cyst cells comprise a single cyst. Spermatogonial cells mature into primary spermatocytes, substantially increase their volume, and undergo meiosis to form spermatids, each of which elongates, matures, and is released as a mature sperm to be stored in the seminal vesicle until copulation. Therefore, a single GSC division has the potential to result in the formation of 64 haploid gametes (Greenspan et al. 2015).

The processes of proliferation and differentiation must be carefully balanced to ensure that each cyst produces the proper number of gametes while avoiding tumor-like overgrowth. In particular, precisely four spermatogonial mitotic divisions must occur to form a normal, 16-cell spermatocyte cyst. Numerous instances have been reported in which these processes fail and result in overproliferation. The genes *bag of marbles (bam), benign gonial cell neoplasm (bgcn)*, and *Rbfox1* act cell-autonomously in the germline, and mutation of these genes results in overproliferation of early, undifferentiated germline cells (McKearin and Spradling 1990; Gonczy et al. 1997; Tastan et al. 2010). In addition, genes affecting signaling, transport, and numerous other processes in the somatic cyst cells have been shown to affect the germline non-autonomously, interfering with the differentiation program and causing dramatic overproliferation of undifferentiated germline cells (Matunis et al. 1997; Kiger et al. 2000; Tran et al. 2000; Joti et al. 2011; Fairchild et al. 2017; Tang et al. 2017). From these studies, a clear picture has emerged in which the close association and signaling relationship between germline and somatic cells is crucial for proper spermatogenesis.

Recent studies have shown that long non-coding RNA transcripts (lncRNAs) are expressed in a tissue-specific manner and are particularly enriched in the testes of Drosophila and other animals, including mammals (Cabili et al. 2011; Young et al. 2012; Brown et al. 2014; Necsulea et al. 2014). Many testis-expressed lncRNAs are enriched in late developmental stages including meiotic and elongated cysts (Wen et al. 2016; Vedelek et al. 2018). Some of them have roles in the late stages of spermatogenesis, as mutations cause spermatid defects including defective chromatin compaction and sperm individualization (Wen et al. 2016).

We are interested in elucidating the effects that non-coding RNAs (ncRNAs), in particular miRNAs, have on processes related to stem cell biology and differentiation and how these processes can be perturbed when miRNAs are dysregulated. It is also important to determine which miRNA target genes are in turn affected in these mutants. To this end, we screened a number of miRNAs reported to exhibit expression in testes and/or ovaries. We closely examined gonads of flies mutant for these miRNAs for phenotypes consistent with stem cell division/self-renewal problems or differentiation defects.

Here we report that deficiency of the miRNA *miR-317* causes the formation of germline tumors in the testes of young flies. Previous reports have implicated *miR-317* in several processes and behaviors, including negative regulation of the Toll immune response (Li et al. 2017; Li et al. 2019); larval ovary morphogenesis and development (Yang et al. 2016); startle-induced locomotion (Yamamoto et al. 2008); aggressive behavioral response (Edwards et al. 2009); female response to mating (Fricke et al. 2014); and regulation of Cyclin B expression during embryonic development (Pushpavalli et al. 2014). However, this work is the first to demonstrate *miR-317* as a tumor suppressor.

The *miR-317* mutant allele *miR-317[ko]* results in the concomitant upregulation of a co-transcribed lncRNA *CR45054* (FBgn0266414), which causes a disruption of the muscle sheath surrounding the testis and a pronounced enlargement of the testis apex. This distinct phenotype causes the testis to resemble the appearance of a peony flower. Consequently, we have named the lncRNA *CR45054* as "*Peony.*" Our data indicate that *miR-317* and *Peony* are mutually expressed from a single polycistronic non-coding RNA locus and their expression depends on differential polyadenylation. Furthermore, we find that overexpression of each of three putative *miR-317* targets, *mastermind, rotund*, or *CG18265*, is sufficient to cause the formation of testis tumors. All three encode transcriptional regulators. Deficiency of *miR-317* results in increased Notch pathway signaling activity in somatic cyst cells; this increase promotes tumorigenesis, as the reduction of Notch activity via gene dose reduction of a crucial Notch cofactor results in a partial abrogation of tumor formation. Our results show that *miR-317* is a novel tumor suppressor, and this important function requires the repression of multiple targets in the somatic cells of the Drosophila testis.

## Results

### *miR-317[ko]* males have multiple testis phenotypes

The Drosophila testis serves as a convenient system for detecting defects in division and maintenance of stem cells as well as the differentiation of their progeny. At the apex of control testes, the region containing transit-amplifying spermatogonia is characterized by bright DAPI staining, owing to the tightly condensed chromatin in these cell types. As germline cells mature into spermatocytes, the cells grow substantially and their chromatin decondenses, leading to much more diffuse DAPI staining (Figures 1C & 1E). In searching for miRNA genes involved in processes of stem cell function and differentiation, we examined the gonads of a loss-of-function (LOF) mutant, *miR-317[ko]*, in which the pre-miRNA has been precisely replaced with a *mini-white* cassette by ends-out homologous recombination (Chen et al. 2014) (Figures 1D, 1F, & 1G). In some *miR-317[ko]* testes, we observed a dramatic enlargement at the testis apex, a phenotype referred to here as apical enlargement (AE; Figure 1D). We also observed an incidence of tumors in *miR-317[ko]* testes, identifiable in adults by very bright DAPI staining apart from the spermatogonial region as well as the strong expression of Vasa, an RNA-binding helicase commonly used as a germline cell marker (Figure 1F).

To reduce the likelihood of genetic background-induced phenotypic artifacts, we wished to analyze the *miR-317* LOF phenotypes using trans-heterozygous allelic combinations. However, aside from *miR-317[ko]* described above, the only *miR-317* mutant stocks available possess large deficiencies that uncover not only the locus of interest, but also many other genes. This is particularly disadvantageous for the analysis of miRNA phenotypes, which tend to be subtle and easily modified by environmental conditions as well as genetic backgrounds (Vidigal and Ventura 2015). Indeed, miRNA mutants often fail to exhibit a phenotype except in a sensitized genetic background (Brenner et al. 2010). Therefore, we generated a new deficiency allele using FLP-mediated recombination between FRT sites present in transposon insertions *PBac{WH}f04665* and *P{XP}d02752* (Parks et al. 2004). This recombination results in a deletion of 5.5 kb that removes only *miR-317* as well as the large second exon of a nearby long non-coding RNA (lncRNA) gene *Peony* (Figure 1G). We predict that this deletion, which we call *Df-miR-317*, represents a LOF allele for both *miR-317* and *Peony*.

We compared testes from *miR-317[ko]* homozygotes and trans-heterozygous (*miR-317[ko]/Df*) adult males and found that testis tumors exist in flies of both genotypes with similar frequency, strongly suggesting that this phenotype is attributable to the loss of the miRNA. In all subsequent experiments requiring *miR-317* LOF, we used either *miR-317[ko]* homozygotes or *miR-317[ko]/Df* flies.

### RNA-seq in *miR-317* mutant testes and ovaries reveals differential expression of genes important for cell signaling and differentiation

To attain a global view of the genes perturbed in *miR-317* mutants, we performed RNA-seq analyses comparing a control genotype, *w[1118]*, to the LOF mutant, *miR-317[ko]*. As the *miR-317[ko]* phenotype indicated a potential issue in germline differentiation, we performed this comparison not only on testes but also on ovaries from adult animals. This allowed us to explore the differences in transcriptional changes between the gonads of both sexes (see Materials and Methods). We detected more total genes in testes than in ovaries (13232 vs. 8894), but we found that more genes are dysregulated in *miR-317* mutant ovaries than in testes, both in number of dysregulated genes and as a percentage of total genes detected: 2944 genes (33.1% of all genes detected) are dysregulated in ovaries, while 1844 genes (13.9% of all genes detected) are dysregulated in testes. In both organs, more genes are downregulated than upregulated in *miR-317* mutants (59.1% in testes, 55.7% in ovaries; Supplemental Tables 1 & 2; Supplemental Figure 1A).

We found that the transcriptional effects of *miR-317* deficiency have both similarities and interesting differences in testes and ovaries. We performed a Gene Ontology (GO)-term enrichment analysis to investigate the biological processes that are overrepresented among the genes dysregulated in both organs. Ovaries and testes share enriched terms pertaining to signal transduction and cell differentiation, as well as cell communication, cell migration, and morphogenesis, all of which terms could be relevant to the tumor phenotype that we discovered in testes. Interestingly, GO terms related to gene expression regulation and cell cycle are highly enriched in mutant ovaries but not testes, while the terms cell junction organization and myofibril assembly are enriched only in testes and not in ovaries (Supplemental Figure 1B).

Consistent with previous reports (Djebali et al. 2012; Brown et al. 2014; Wen et al. 2016; Joshi and Rajender 2020; Hong et al. 2021), we detected a substantially higher proportion of lncRNA genes in testes than in ovaries (Supplemental Tables 1 & 2; Supplemental Figure 1A). In contrast to protein-coding genes, lncRNAs in both organs are more likely to be up- than downregulated. This is particularly striking in the testes, in which the five most-upregulated genes, and more than a third of the top 100 most-upregulated genes, are ncRNAs (Supplemental Table 1). Among those lncRNA genes strongly upregulated in *miR-317[ko]* testes and ovaries is *Peony*, the gene just upstream of *miR-317* (Supplemental Figure 1C; Supplemental Tables 1 & 2).

Interestingly, we noted that while *miR-317[ko]* and *miR-317[ko]/Df* testes exhibit an apical enlargement (AE) phenotype at similar rates, we do not observe this phenotype in *Df-miR-317* testes. As the primary difference between *miR-317[ko]* and *Df-miR-317* is the deletion of *Peony* only in the latter, this discrepancy raises the possibility that the lncRNA is involved in the development of the AE phenotype. We decided to look first into the roles of the *Peony* lncRNA in testes phenotypes, and then at the role of *miR-317* as a tumor suppressor. In spite of the large number of genes and important processes that are disrupted in *miR-317* mutant ovaries, we did not observe any incidence of tumors or other remarkable phenotypes similar to those found in testes. Therefore, we opted to focus the rest of our study on testes.

### Upregulation of *Peony* causes disruption of the testis muscle sheath

**Figure 2.**
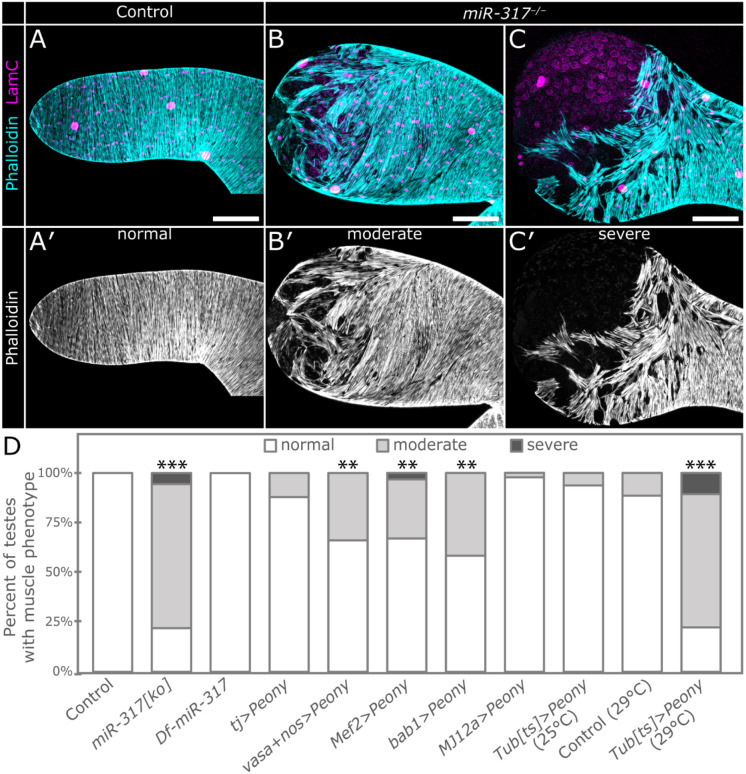
Upregulation of Peony causes a disruption of the testis muscle sheath **A** Control testis apex stained with Phalloidin (cyan) to mark the muscle sheath and anti-LamC (magenta) to mark nuclear envelopes. Phalloidin is shown alone in grayscale in **A′**. Multinucleated muscle cells have many small nuclei; pigment cells have large, sparsely spaced nuclei. **B-C** *miR-317*^−/−^ testis apexes with moderate (**B, B′**) and severe (**C, C′**) muscle sheath disruption. In C, the muscle sheath is severely disrupted, and the underlying germline is revealed by nuclear envelope staining (LamC, magenta). **D** Quantification of muscle disruption phenotypes in testes of various genotypes: *miR-317* mutants and *Peony* overexpression using cell- and tissue-specific drivers. **, p<10^−3^; ***, p<10^−9^ as determined by chi-square analysis. Scale bars: 100 μm.

Analyses of mutant testes revealed that AE is concomitant with a disruption of the muscle sheath that surrounds the testis (Figures 2A-C). While nearly 80% of *miR-317[ko]* testes exhibit some degree of muscle disruption, *Df-miR-317* testes muscles appear intact as in controls (Figure 2D). Since *miR-317* mutants have significantly upregulated *Peony* (Supplemental Tables 1 & 2), we generated a *UAS-Peony* transgene to allow conditional overexpression of a full-length cDNA. In order to determine whether *Peony* plays a role in the AE or muscle disruption phenotype, we overexpressed *Peony* using Gal4 lines that express in various cell types in the testes (Supplemental Figure 2). Indeed, we found that muscle disruption is evident upon *Peony* overexpression in some cell types. Expression of *Peony* in somatic cells or germline cells – using *tj-Gal4* or *vasa-Gal4+nos-Gal4*, respectively – causes muscle disruption in 13-34% of testes (Figure 2D). Consistent with *Peony* having a role in the muscle disruption phenotype, overexpression with *Mef2-Gal4*, expressed in muscle cells, causes muscle sheath disruption in 42% of testes, while overexpression with *MJ12a-Gal4*, which drives expression only in the pigment cell layer that also surrounds the testis (Hrdlicka et al. 2002) (Supplemental Figure 2), does not cause muscle disruption (Figure 2D). Overexpression using *bab1-Gal4*, expressed in somatic cyst cells, muscle cells, and pigment cells, causes muscle sheath disruption in 42% of testes.

Since none of the conditions described above result in a muscle disruption phenotype as severe as *miR-317[ko]*, we turned to ubiquitous overexpression using *Tub-Gal4*. This condition causes a high degree of lethality, so we used a *Tub-Gal4; Tub-Gal80[ts]* combination (*Tub-Gal4[ts]*) to block expression until pupal stages. This results in a strong incidence of muscle disruption very similar to that seen in *miR-317[ko]* testes (Figure 2D). Moreover, while overexpression using the other cell-type-specific drivers failed to result in AE, this phenotype does manifest upon ubiquitous overexpression. Increased *Peony* levels did not, however, result in the formation of tumors with any driver. We conclude that while tumor formation is due to a loss of *miR-317* function, the AE phenotype is a result of the upregulation of *Peony* that occurs in *miR-317[ko]* testes or upon exogenous overexpression.

### *miR-317* and *Peony* comprise a single transcription unit

**Figure 3.**
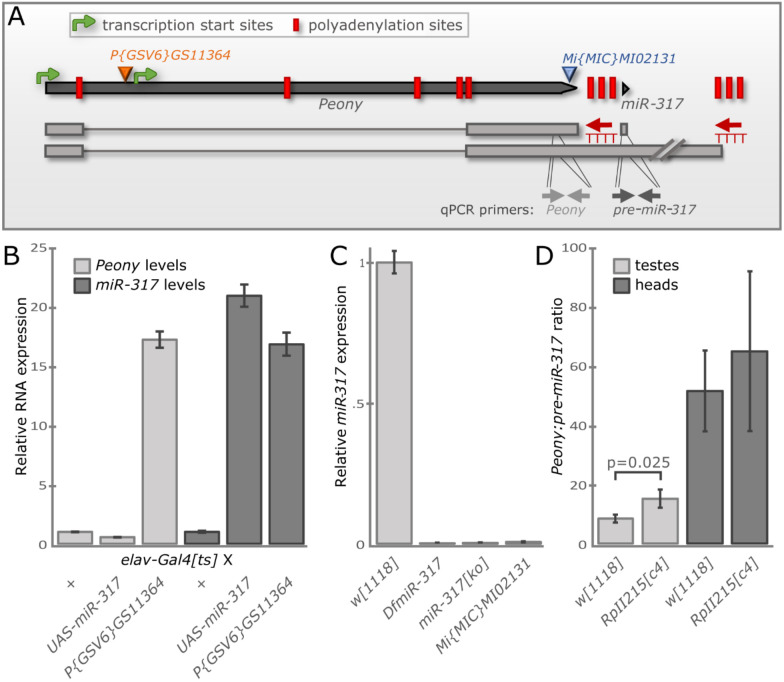
*Peony* and *miR-317* comprise a bicistronic transcript and are regulated by alternative polyadenylation **A** *Peony-miR-317* locus showing transposon insertion sites and predicted and annotated transcription start sites and polyadenylation sites. The most distal polyadenylation sites are not annotated and are predicted based on this work. Transcripts (light gray rectangles) depict putative alternative isoforms that differ based on alternative polyadenylation. **B** Results of qRT-PCR using the qPCR primers shown in A showing relative *Peony* and pre-*miR-317* levels in control and overexpression conditions. Overexpression was performed using the driver *elav-Gal4; Tub-Gal80[ts] (elav-Gal4[ts]*). **C** Mature *miR-317* levels in control (*w[1118]*) and three mutant genotypes: *Df-miR-317, miR-317[ko]*, and *Mi{MIC}MI02131*. **D** *Peony:*pre-*miR-317* ratios resulting from qRT-PCR using the qPCR primers shown in A in two tissues (heads and testes) from two genotypes: control (*w[1118]*) and *RpII215[c4]*. Statistical significance determined using a Student’s t-test.

The close proximity of *Peony* to *miR-317* (Figure 3A) and its strong upregulation in *miR-317[ko]* mutant gonads led us to investigate how their expression is interrelated. We first hypothesized that the upregulation of *Peony* in *miR-317[ko]* testes may be due to direct targeting of the lncRNA by *miR-317*. We examined the sequences of all 192 lncRNAs dysregulated in mutant testes for the presence of *miR-317* binding sites. Of the 140 upregulated lncRNA genes, 38 (27%) have predicted target sites; indeed, this includes the *Peony* transcript. Among the 52 downregulated lncRNA genes, 18 (35%) have predicted binding sites (Supplemental Table 1). Thus, the presence of a putative *miR-317* binding site does not appear to be predictive of a lncRNA’s upregulation in the absence of *miR-317*. Furthermore, the overexpression of *miR-317*, which results in a strong upregulation of the miRNA itself, results in only a small reduction in *Peony* expression (Figure 3B). We conclude that the regulation of *Peony* is likely not due to direct targeting by *miR-317*.

Alternatively, the two genes may be co-transcribed, consistent with a report that proposed that *Peony* indeed serves as the primary miRNA transcript from which *miR-317* is processed (Kadener et al. 2009). In this case, either the *mini-white* present in *miR-317[ko]* or the lack of miRNA hairpin may cause the transcript to be either upregulated transcriptionally or less efficiently degraded, causing *Peony* accumulation. To investigate this possibility, we acquired an independent UAS insertion in the *Peony* gene, *P{GSV6}GS11364*, inserted in the intron (Figure 3A). We crossed this line to a Gal4 driver to test its capacity to induce expression of downstream sequence; we used the pan-neuronal driver *elav-Gal4* for this purpose because it drives expression in thousands of cells in the larval central nervous system, allowing the ready acquisition of a large amount of RNA for RT-qPCR. Overexpression of *miR-317* using *elav-Gal4* causes larval lethality; therefore, we used a temperature-sensitive inhibitor of Gal4, Gal80[ts], to block expression during early developmental stages and to allow larvae to survive until the late L3. We found that *P{GSV6}GS11364* causes upregulation of *Peony* sequence downstream of the insertion sites. Notably, Gal4-induced overexpression from this insertion in *Peony* also causes the upregulation of *miR-317*, demonstrating that the two non-coding RNAs can exist in a single transcript (Figure 3B). Consistent with this, three additional UAS-containing transposons, each inserted in the second exon of *Peony*, are able to promote similar upregulation of downstream sequences, including *miR-317* (Supplemental Figures 3A-B).

Moreover, we tested the expression level of *miR-317* in *miR317[ko]* and *Df-miR-317* flies, as well as in flies homozygous for another transposon insertion in *Peony, Mi{MIC}MI02131*, which contains a transcriptional stop signal. As expected, *miR-317* is undetectable in the two null alleles. We also found that *Mi{MIC}MI02131* causes a strong loss of *miR-317* expression (Figure 3C), which would only be expected if *miR-317* were obligatorily co-expressed as part of the *Peony* transcript. Consistent with this prediction, we observe that while a transcription start site (TSS) is predicted near the 5' end of *Peony*, there is no independent TSS predicted upstream of the miRNA (Figure 3A). We conclude that and that *Peony* is the primary miRNA from which *miR-317* is processed.

It has been suggested that *miR-317* is expressed as a cluster with two downstream miRNA genes, *miR-277* and *miR-34*, both of which are processed from the ncRNA gene *CR43459* (Kadener et al. 2009; Zhou et al. 2018). Our data suggest that this is not the case, at least in testes and ovaries. First, although our RNA-seq data demonstrate that *Peony* is strongly upregulated in *miR-317[ko]* testes and ovaries, expression levels of *CR43459* do not change in either organ (Supplemental Tables 1&2). Additionally, a TSS has been identified at the beginning of each of these ncRNA genes, *Peony* and *CR43459*, consistent with their independent transcription in spite of their close proximity.

It has been shown in plants that small regulatory peptides encoded by otherwise non-coding RNAs can act in a feedback mechanism to upregulate their parent transcript (Lauressergues et al. 2015). While *Peony* is predicted to be a ncRNA, its sequence includes an open reading frame that would encode an 83 amino acid peptide if translated. To determine whether this putative peptide, or indeed the *Peony* transcript itself, is involved in regulation of its own expression, we overexpressed *Peony* in larval brains using our UAS transgene (*elav>Peony*). Since the *UAS-Peony* transgene was created using a full-length cDNA (see Materials and Methods), qPCR primers within the transcript sequence could not be used to assay expression levels of the endogenous gene; therefore, we made use of *Mi{MIC}MI02131*, which is inserted in exon 2 of *Peony* as described above (Figure 3A) and is thus included in a truncated *Peony* transcript. Using primers against the GFP coding sequence within the MiMIC transposon, we performed RT-qPCR on larval brains with or without the overexpression of *Peony*. We found that, as expected, *GFP* expression is absent from brains lacking the MiMIC insertion and present in those containing MiMIC. Importantly, *GFP* expression does not change upon overexpression of *Peony* (Supplemental Figure 3C). This indicates that the increased expression of *Peony* does not affect transcription at its endogenous locus and that no feedback mechanism is involved in its regulation.

### *miR-317* levels depend on alternative polyadenylation of the *Peony* transcript

It is unclear if *miR-317* is constitutively co-transcribed, or whether *Peony* may sometimes be expressed independently. Three polyadenylation sites (PASs) are predicted between *Peony* and *miR-317* (RegRNA 2.0) (Chang et al. 2013), indicating that in some circumstances, transcription may terminate before the expression of the miRNA (Figure 3A). This raises the interesting possibility that the differential usage of PASs may play a role in regulating the expression of *miR-317*.

To test this, we first sought to determine whether a distal, heretofore unannotated PAS exists downstream of the miRNA. For these experiments, we decided to compare adult testes to heads because numerous studies have demonstrated that the process of polyadenylation is regulated differently in brains than in other somatic tissues (Beaudoing and Gautheret 2001; Smibert et al. 2012; Yatsenko et al. 2014; MacDonald 2019). We reasoned that using heads allows the strong enrichment of brain-expressed RNAs. We extracted RNA from testes and heads of male *w[1118]* flies, completed reverse transcription (RT) using an oligo-dT reverse primer, and used the resulting cDNA to perform PCR using primers specific to the pre-miRNA locus. This procedure yields PCR product detectable in RT-qPCR experiments (see below), which would only be expected if there is indeed a PAS downstream of the miRNA.

Next, we sought to determine whether differential polyadenylation regulates the expression of *miR-317*. First, we again performed RT using oligo-dT, this time followed by qPCR using two sets of primers: one targeting the pre-miRNA region and another in the second exon of *Peony* (Figure 3A). In this way, we were able to quantify both regions of the parent transcript and calculate a *Peony:miR-317* ratio for different tissues. Comparing RNA extracted from testes and male heads, we found a striking difference in the ratio between these tissues (52:1 ratio in heads vs 8.5:1 in testes; Figures 3D). This result shows that *Peony* abundance is much greater than that of *miR-317* and that their relative expression levels vary widely between different tissues. We propose that alternative PASs exist: a proximal site between the two genes and a distal site downstream of the miRNA (Figure 3A). The differential expression of the RNAs may result either from a propensity for the transcription machinery to utilize the proximal site, the rapid processing of the miRNA hairpin and subsequent degradation of the miRNA-containing transcript, or both.

To further test whether differential polyadenylation plays a substantial role in the observed prevalence of the *Peony* transcript, we made use of *RpII215[c4]*, an RNA polymerase II subunit mutation that results in reduced transcription elongation speed. This slowed elongation imparts to the transcription apparatus the tendency to utilize a proximal PAS (Liu et al. 2017). In the context of coding genes, the choice of a proximal or a distal PAS usually results in a transcript with a shorter or a longer 3'UTR, respectively. We sought to test whether forcing a proximal PAS bias results in an increased expression of *Peony* at the expense of *miR-317*. We compared *Peony:miR-317* ratios in male heads and testes between *w[1118]* control flies and *RpII215[c4]* homozygotes. In male heads, we found that this ratio does not change significantly in the slow elongation mutant (Figure 3D). In contrast, the ratio of *Peony:miR-317* increases nearly 2-fold in *RpII215[c4]* testes, from 8.5 to 15.3 (Figure 3D). Consistent with our observations, while the *RpII215[c4]* mutant is known to enhance the usage of proximal PASs in Drosophila bodies, this phenomenon was not observed in heads (Liu et al. 2017). It is thought that this is due to additional stringent controls regulating 3'UTR length in the brain. These results strongly suggest that, in testes, the relative levels of *Peony* and *miR-317* are affected by the differential choice of either a proximal or a distal PAS.

### *miR-317* LOF tumors consist of GSC-like and early spermatogonial cells

**Figure 4.**
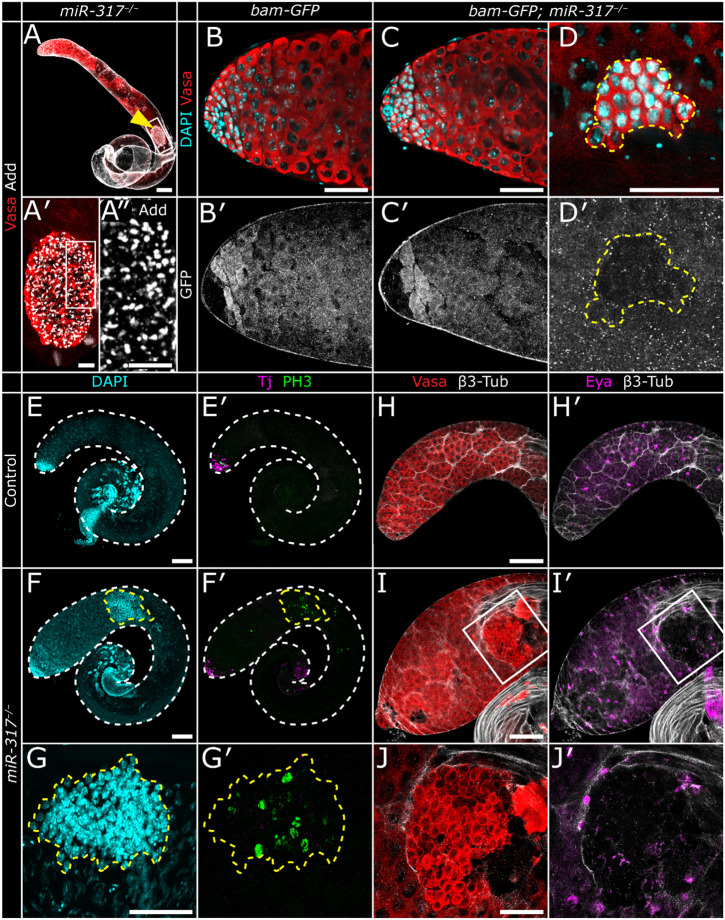
*miR-317* mutant testis tumors are composed of early germline cells **A** *miR-317*^−/−^ testis exhibiting a tumor (yellow arrowhead) with strong Vasa (red) and Adducin (Add; grayscale) staining. **A′** Enlargement of the tumor outlined with a white rectangle in A. All cells are Vasa-positive and Add-positive. **A″** Enlargement of the white rectangle in A′ shows Add staining exists mostly in spherical-shaped spectrosomes, with a minority exhibiting minimal branching. **B** Apex of testis with a *bam* transcriptional reporter (*bam-GFP*). GFP is shown in single panel in B′. **C** *bam-GFP* in a *miR-317*^−/−^ background; GFP pattern (**C′**) does not appear different from control (**B′**). **D** Tumor in a *bam-GFP; miR-317*^−/−^ testis; tumors of this genotype do not express *bam-GFP* (**D′**). **E** Control testis (outlined with white dashed line) exhibiting strong DAPI staining near the apex, in the region where germline cells undergo mitotic divisions. **E**′ Tj-positive cyst cells reside near the testis apex, in proximity of the mitotic zone. PH3-positive cells mark mitotically active germline cells, which divide in synchrony within each germline cyst. **F** *miR-317*^−/−^ testis with a large tumor, outlined by yellow dashed line and identified by region of bright DAPI-positive cells removed from the apex. **F**′ Same testis as shown in F, now showing that while some cells in the tumor are PH3-positive, the Tj-positive cells are present only near the apex as in controls. **G** A *miR-317*^−/−^ tumor with bright DAPI staining. **G**′ Same tumor as shown in G. Some scattered cells are PH3-positive; tumor and surrounding cells are Tj-negative. (E,F, & G: DAPI, cyan; PH3, green; Tj, magenta). **H** Apex of control testis. Vasa marks germline cells and β3-Tub marks cyst cell cortexes. **H**′ Same testis as in H; Eya marks somatic cyst cell nuclei. **I** *miR-317*^−/−^ testis stained with Vasa and β3-Tub and exhibiting a tumor (indicated by white box). **I**′ Same testis as in I; β3-Tub and Eya mark cyst cell cortexes and nuclei, respectively. **J-J**′ Enlargement of the tumor shown in I & I′ showing that while Vasa is strongly expressed in the tumor, β3-Tub and Eya are excluded. (H-J: Vasa, red; β3-Tub, grayscale; Eya, magenta). A, E, F, H, I: scale bars 100 μm. B, C, D, G, & J: scale bars 50 μm. A’, A”: scale bars 20 μm.

Next, we turned our attention from the lncRNA, *Peony*, to examine the tumor suppressive functions of *miR-317*. To more fully characterize the *miR-317[ko]* testis tumor phenotype, we stained control and mutant testes with antibodies that mark different cell types and indicate different stages of differentiation. The strong expression of Vasa and Adducin (Add) suggests that the tumors are largely composed of germline, not somatic cells (Figures 4A & 4A'). We used several additional means to verify this. Add interacts with the actin cytoskeleton and labels distinct germline-specific structures in early germline cells: spectrosomes and fusomes. The morphology of these structures can indicate the identity (i.e., differentiation state) of these cells: spherical spectrosomes are indicative of a GSC or gonialblast (the immediate daughter of a GSC) identity, while branched fusomes indicate a more mature spermatogonial identity. We found that the tumors in *miR-317* mutants possess mostly spectrosomes but can also contain a smaller amount of branched fusomes, sometimes within a single tumor (Figure 4A"). These results demonstrate that the majority of the cells within the tumors have an early germline identity.

The pro-differentiation protein Bam is an indicator of the differentiation state of germline cells. In control testes, Bam is expressed in germline cells of 4- to 8-cell cysts and is absent from GSCs, gonialblasts, and 2-cell cysts. *bam-GFP* is a reporter construct consisting of *bam* regulatory elements driving the expression of GFP (Chen and McKearin 2003). As a result, GFP is expressed in the endogenous *bam* pattern: excluded from the most apical germline cells but expressed in 4- and 8-cell cysts. Unlike Bam protein, GFP perdures into later spermatogonial and spermatocyte stages (Figures 4B-4B'). Deficiency of *miR-317* does not change this pattern of GFP expression (Figures 4C-4C'). We found that *bam-GFP* is not expressed in *miR-317[ko]* GLTs (Figures 4D-4D'), indicating that in terms of differentiation state, the germline cells comprising the tumor are earlier than the spermatogonia of 4-cell cysts. Taken together, our data suggest that *miR-317[ko]*-derived GLTs consist of early, undifferentiated germline cells similar to GSCs, gonialblasts, or 2-cell cysts. Next, we wanted to determine whether somatic cells also contribute to the cellular composition of the tumors.

The soma-expressed transcription factor, Traffic jam (Tj), is expressed in CySCs and early cyst cells near the testis apex in control and *miR-317* mutants but is not found in cells in or around the tumors in *miR-317[ko]* testes (Figures 4E'-4G'). To investigate whether tumor cells are proliferative in adult testes, we stained for the mitotic marker, phosphorylated histone H3 (PH3). The spermatogonial cells within each normal cyst divide synchronously in clusters of 2, 4, or 8 cells. We found that while some cells in each tumor are PH3-positive, demonstrating that they are mitotically active, most of the dividing cells are dispersed, not clustered (Figure 4F'-4G'). This indicates that tumors consist of cells that divide similarly to GSCs or early spermatogonia.

We also stained testes with multiple other somatic cell markers. Eyes absent (Eya) is a transcription factor expressed in mature somatic cyst cells, while β-3-Tubulin marks the cortexes of somatic cells and thus nicely outlines differentiating cysts (Figures 4H-H'). We found that Eya-positive somatic cells can be found adjacent to tumors, but not within them; similarly, tumors express β-3-Tubulin at their periphery, but this somatic marker does not extend within the tumor (Figures 4I-J'). Our results lead us to conclude that *miR-317* mutant testis tumors consist not of soma but of germline cells, probably a mix of cells similar in appearance to GSCs and their spermatogonial progeny. Hereafter we refer to these tumors as germline tumors (GLTs).

### GLTs arise developmentally and disintegrate in aging testes

**Figure 5.**
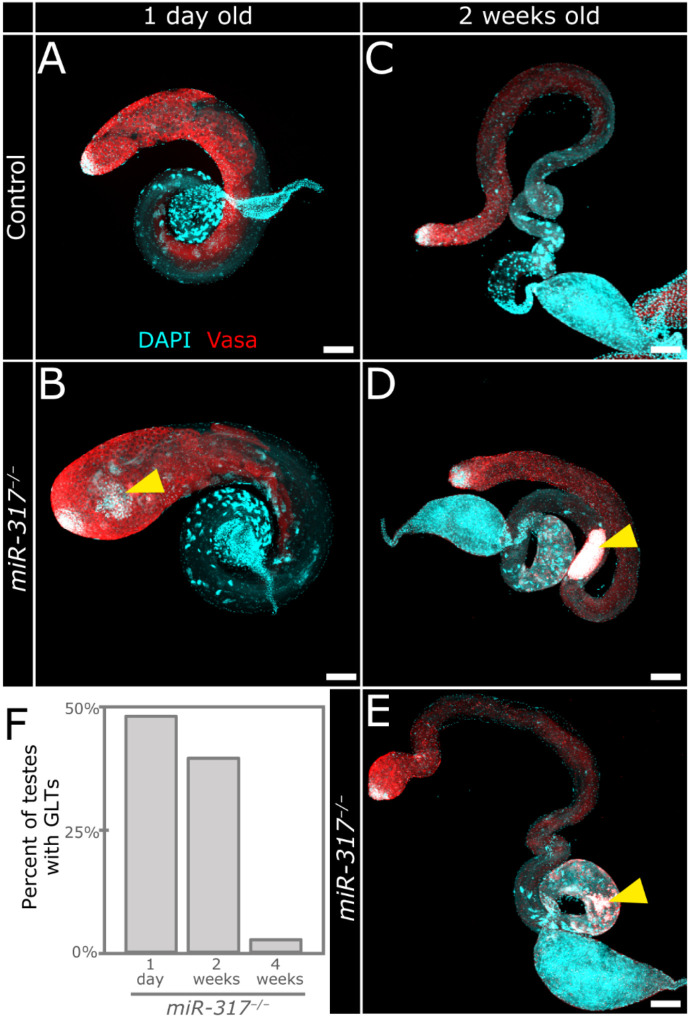
GLTs arise near the testis apex and disintegrate basally in aging testes **A** Young, 1-day-old control testis stained for DAPI (cyan) and Vasa (red). **B** Young *miR-317*^−/−^ testis exhibiting a GLT near the apex (yellow arrowhead) with strong Vasa and DAPI staining. **C** 2-week-old control testis. **D** 2-week-old *miR-317* mutant testis with a large GLT in the basal half of the testis (yellow arrowhead). **E** A different 2-week-old *miR-317* mutant testis with the partially disintegrated remains of a GLT near the base of the testis (yellow arrowhead), discernible by strong Vasa staining, which normally does not occur in this region. **F** Graph showing the incidence of GLTs in *miR-317*^−/−^ testes over time. Scale bars 100 μm.

As our investigations of the GLT phenotypes described above were limited to young, 0-3-day-old males, we next sought to determine whether the GLT phenotype persists in aging flies. We dissected 1-day-old, 2-week-old, and 4-week-old males. Control males never exhibit GLTs (Figures 5A & 5C). In young *miR-317[ko]/Df* males, as described above, we found GLTs near the apexes of testes (Figure 5B). While in 2-week-old flies, testes still exhibit the presence of GLTs, they are usually not found at the apical end of testes; rather, most of them exist midway down the testis toward the posterior end (Figure 5D). In addition, we found evidence of the fragmented remains of tumors near the testis base just apical to the seminal vesicles, evidenced by clusters of small cells stained very strongly with Vasa (Figure 5E), a characteristic never seen near the base of control testes (Figure 5C). In older, 4-week-old testes, we found a pronounced decrease in the incidence of GLTs, indicating that new tumors do not form in aging, adult flies (Figure 5F). We conclude that GLTs in *miR-317* LOF testes originate from germline cells near the hub and then migrate posteriorly through the testis, finally disintegrating near the testis base. Furthermore, we find that the formation of these GLTs is a phenomenon that occurs during development and in very young flies but does not persist in aging, adult males.

### Relevant *miR-317* targets involved in mutant phenotypes

To understand the molecular mechanism of *miR-317* involvement in GLT tumors, we utilized our RNA-seq data to identify candidate *miR-317* target genes in testes. We used multiple online databases to compile a list of over 1000 predicted direct targets (see Materials and Methods). 919 of these predicted targets were detected in our testis RNA-seq data, of which 142 are significantly dysregulated in *miR-317[ko]* testes (p<.05; 88 downregulated and 54 upregulated; Supplemental Table 3). Genes dysregulated in *miR-317[ko]* are expected to include both direct *miR-317* targets and indirectly affected, downstream genes. For this reason, we placed the highest priority on upregulated genes because we expect transcripts that are directly targeted by *miR-317* to be upregulated in the mutant. Applying an even more stringent requirement that a gene should be upregulated with at least a Log_2_ fold change (Log_2_FC) of 0.5 reduces the list of upregulated genes from 54 to 15 genes; we propose that these 15 genes are strong candidates to be direct targets of *miR-317* in testes (Table 1). We further investigate these genes’ expression and involvement in mutant phenotypes below.

**Figure 6.**
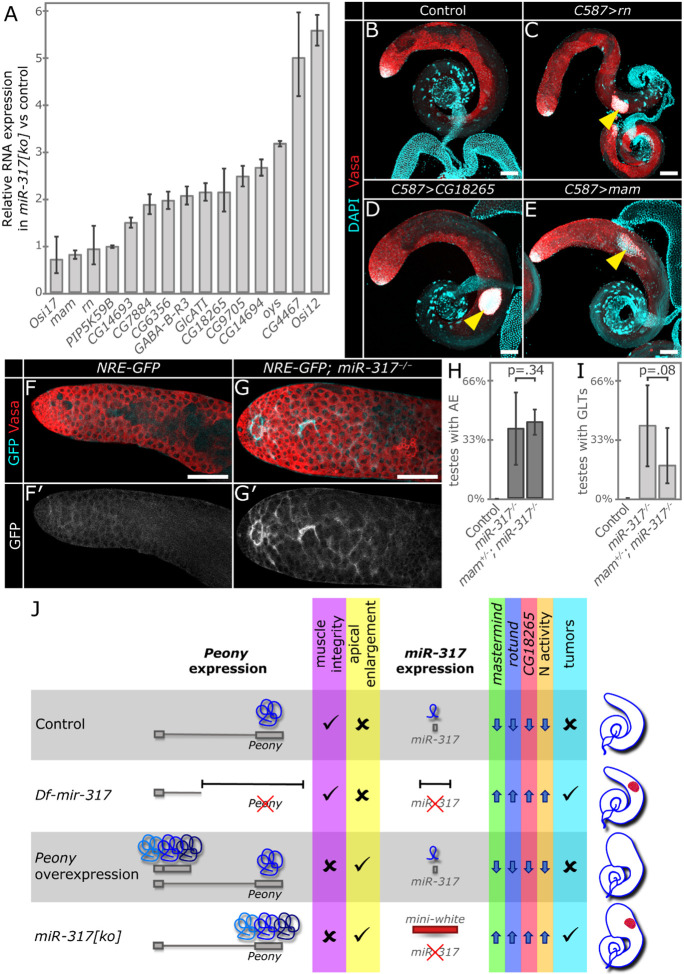
*miR-317* regulates multiple targets to prevent tumor formation **A** qRT-PCR quantification of putative *miR-317* target genes. **B** Control testis stained for DAPI (cyan) and Vasa (red). **C-E** Testis overexpressing *rn* (C), *CG18265* (D), and *mam* (E) under the control of the cyst-cell-specific driver *C587-Gal4*, each exhibiting a GLT (yellow arrowhead). **F** Apex of control testis (*NRE-GFP*) in which GFP is expressed under the control of a Notch reporter element (NRE). GFP is nearly undetectable (GFP shown in grayscale in F′). **G** GFP expression is strongly upregulated in *NRE-GFP; miR-317*^−/−^ testes (GFP shown in grayscale in G′). **H-I** Quantification of the percentage of testes that exhibit GLTs (H) or AE (I) in control, *miR-317*^−/−^, or *mam*^+/−^; *miR-317*^−/−^ testes. Statistical significance assessed using the Student’s t-test. Scale bars 100 μm. **J** Summary of our proposed model of the differential effects of *Peony* and *miR-317* on testes. In controls, *Peony* is expressed at its endogenous level, muscle integrity is intact, and AE is absent; *miR-317* is expressed, repressing target genes *mam, rotund*, and *CG18265* and Notch (N) activity, and tumors are absent. In *Df-miR-317* and *miR-317[ko], miR-317* is absent, target genes are upregulated, N activity is increased, and GLTs (red clusters in testis schematics) form. In both *Peony* overexpression and *miR-317[ko], Peony* levels are high, causing loss of muscle integrity and the appearance of AE.

We first sought to confirm the upregulation of these genes in testes using reverse-transcriptase quantitative PCR (RT-qPCR). We used *αTub84B* as an endogenous control, expression of which is not changed in *miR-317* mutant testes or ovaries (Supplemental Tables 1 & 2). By comparing the expression levels of putative target genes between *w[1118]* and *miR-317[ko]* (the genotypes used for RNA-seq analysis), we confirmed the upregulation of most of the genes in mutant testes (Figure 6A).

We next sought to recapitulate *miR-317[ko]* mutant phenotypes via the upregulation of candidate targets of *miR-317*. For seven of these genes, we were able to acquire Drosophila stocks predicted to cause Gal4-induced upregulation (Table 2). These stocks were either in vitro-cloned UAS lines, UAS-containing transposon insertions, or CRISPR-OE lines. The latter is a stock that ubiquitously expresses a guide RNA (gRNA) specific for a gene of interest; when crossed to a stock containing both an appropriate Gal4 driver and *UAS-dCas9.VPR*, a construct expressing a nuclease-dead Cas9 protein fused with a transcriptional activation domain, this can cause the overexpression of the gene of interest from its endogenous locus (Lin et al. 2015).

For germline overexpression, we used *nos-Gal4*, which is expressed in GSCs and early germline cells, and for somatic overexpression, we used *C587-Gal4*, which is expressed in cyst stem cells and cyst cells (Kawase et al. 2004) (Supplemental Figure 2). First, we overexpressed in germline cells those genes with UAS lines available and were unable to detect the AE phenotype or the formation of GLTs (Table 2). Next, we overexpressed in somatic cells using either *C587-Gal4* or *C587-Gal4; UAS-dCas9.VPR*, as appropriate (Table 2). While overexpression of several of the candidate genes causes no discernible testis phenotypes, somatic expression of either *rotund (rn)* or *CG18265* is sufficient to cause GLTs (Table 2; Figures 6B-D). The AE phenotype was not observed in any of the overexpression experiments. Both *rn* and *CG18265* encode transcriptional regulators with no previously known roles in testes or tumorigenesis. We conclude that overexpression of multiple putative *miR-317* target genes can contribute to the formation of GLTs in the testis and propose that in wild-type testes, the miRNA *miR-317* is likely active in repression of multiple targets in its role as a tumor suppressor.

### The Notch cofactor Mastermind is required for the formation of GLTs

Among the genes dysregulated in *miR-317[ko]* testes are many interactors and regulators of Notch signaling (Supplemental Table 4). Among these genes is *mastermind (mam)*, which encodes a transcriptional co-factor in canonical Notch signaling. *mam* is a predicted target of *miR-317* (Supplemental Table 3), although it was not upregulated in our RNA-seq dataset (Supplemental Tables 1 & 2). We overexpressed *mam* in somatic cells using *C587*-Gal4 and found that this condition results in formation of GLTs but not AE (Figure 6E). As Mam is an important positive Notch regulator, this result suggests that strengthened Notch signaling in somatic cyst cells may be a driver of tumorigenesis.

To address the involvement of Notch in GLT formation, we next attempted to augment Notch signaling by overexpressing other components of Notch signaling. We performed germline overexpression of the Notch ligand Delta or somatic overexpression of multiple forms of activated Notch. We also overexpressed Notch clonally using the Flp-out method (Struhl and Basler 1993). While Notch overexpression consistently causes a high degree of lethality, none of these conditions is sufficient to cause the formation of GLTs. However, overexpression of *mam* in Flp-out clones results in tumors, similar to its somatic overexpression (Table 2).

The involvement of Mam in GLT formation as well as the dysregulation of many Notch regulators led us to further investigate a potential involvement of the Notch pathway in *miR-317* LOF phenotypes. We sought to determine whether Notch signaling strength is affected by *miR-317* LOF, making use of a reporter in which GFP is under the transcriptional control of a Notch responsive element (*NRE-GFP*) (Saj et al. 2010). We found that the GFP signal is nearly undetectable in most control testes from young, 1-day-old flies. In those that express detectable GFP, the pattern is consistent with weak Notch signaling activity in cyst cells (Figures 6F-F'). Upon *miR-317* LOF, GFP expression is strongly upregulated in mature cyst cells; GFP signal remains undetectable in the cells closest to the apex (Figures 6G-G'). We conclude that *miR-317* normally acts to repress Notch signaling in cyst cells, probably in part by regulation of *mam* expression.

Finally, we tested the necessity of increased Mam for either the GLT or the AE phenotype by introducing a strong *mam* LOF allele, *mam[8]*, into the *miR-317[ko]* background. We found that the loss of one copy of *mam* fails to suppress the AE phenotype (p=0.34), suggesting that this phenotype is not due to increased Notch signaling (Figure 6H). In contrast, the incidence of GLTs is marginally suppressed (p=0.08) by *mam* heterozygosity (Figure 6I). This result suggests that increased Notch signaling strength is required for GLT formation in *miR-317* mutant testes. Taken together, our data strongly suggest that Mam is both necessary and sufficient for testis GLT formation, and that *miR-317* represses *mam* and multiple other transcripts in its tumor-suppressive capacity.

Our data demonstrate that *Peony* and *miR-317* have separable effects: upregulation of the *Peony* transcript causes lesions to appear in the testis muscle sheath leading to AE, and loss of *miR-317* causes upregulation of *CG18265, mam*, and *rn* and increased Notch signaling strength, promoting the formation of GLTs (Figure 6J). Consistent with these conclusions, overexpression of *CG18265, mam*, or *rn* causes the formation of germline tumors but does not lead to muscle breakage or apical enlargement. Ubiquitous overexpression of *Peony* from our inducible transgene does not cause GLT formation but results in a testis muscle breakage phenotype as well as a concomitant apical enlargement similar to that seen in *miR-317[ko]* testes (Figure 6J).

## Discussion

### *miR-317* regulates multiple genes to repress tumor formation in testes

It has become clear in recent years that a miRNA or miRNA cluster can target multiple genes in the same pathway to ensure a speedy and robust response (Mestdagh et al. 2010; Uhlmann et al. 2012; Cicek et al. 2016; Hart et al. 2019). We report here that repression of each of three putative *miR-317* targets, *mam, rn*, and *CG18265*, is necessary to prevent the onset of tumors in Drosophila testes. All three genes encode transcriptional regulators, of which Rn and the CG18265 gene product are C2H2 zinc-finger transcription factors, and Mam is well known as a transcriptional co-factor in the regulation of Notch target genes. Interestingly, Rn is also reported to affect Notch signaling in multiple contexts: *rn* mutation dominantly enhances the *split* phenotype caused by a recessive *Notch* allele (Brand and Campos-Ortega 1990); and the expression patterns of the Notch ligands Delta and Serrate are perturbed by *rn* mutation in imaginal discs (St Pierre et al. 2002). No link between *CG18265* and Notch signaling has yet been demonstrated.

Rn has important roles in olfactory neuron specification and imaginal disc development. While not an essential gene, both male and female *rn* mutants are sterile. This sterility is not germline-dependent (Kerridge and Thomas-Cavallin 1988), suggesting that Rn has a role in the somatic gonadal cells. While it is not known why *rn* mutants are sterile, our findings suggest that *miR-317* acts in somatic cells, at least in the testes, to modulate the levels or expression pattern of *rn* to prevent germline overproliferation. Notably, *rn* is not dysregulated in ovaries per our RNA-seq data (Supplemental Table 2), implying that it is not similarly affected by *miR-317* in both organs.

### Sufficiency of Mam and Notch in driving GLT formation

We show in the present study that overexpression of Mam in cyst cells is sufficient to cause GLT formation, and reduction of Mam partially abrogates the incidence of GLTs. Although Mam has been well-studied as an important transcriptional co-factor in canonical Notch signaling, it has also been reported that in various contexts, Mam functions in a Notch-independent manner (Kankel et al. 2007; Vied and Kalderon 2009; Zhang et al. 2011; Lobo-Pecellin et al. 2019). While a Notch-independent Mam function remains a possibility in the context of testis GLT formation, we believe this is unlikely in light of our finding that *miR-317[ko]* causes increased Notch activity in cyst cells, the same cell type in which Mam overexpression results in GLTs. Thus, it is likely that cyst cell-expressed Mam participates in this Notch signaling.

While we were unable to show that overexpressed Notch drives GLT formation, the possibility remains that increased Notch signaling may drive tumorigenesis in conditions that we did not test. Further manipulation of the timing and strength of the Notch pathway would be required to truly exclude this possibility; additionally, a more detailed exploration of the genetic interaction between *Notch* and *miR-317* should prove informative. Alternatively, Notch overexpression may not induce GLTs if Mam is a limiting factor in determining the strength of Notch signaling. In this instance, reducing the gene copy number of *mam* decreases the incidence of tumors as we demonstrate here, but increasing Notch without simultaneously increasing Mam is insufficient to induce tumor formation. Consistent with this possibility, in Drosophila ovaries, somatic overexpression of activated Notch and Mam simultaneously results in tumor-like overgrowth, while expression of Notch alone does not (Defreitas et al. 2020).

### Increased Notch signaling: differential effects depending on cyst developmental stage?

Intriguingly, the consequences of Notch involvement in various processes differ in a cell- and context-dependent manner. For instance, Notch can behave either as an oncogene or as a tumor suppressor, depending on the cell type; similarly, either proliferation or terminal differentiation can be promoted by increased Notch signaling (Koch et al. 2013; Yatsenko and Shcherbata 2018; Yatsenko and Shcherbata 2021; Zamfirescu et al. 2022). In Drosophila, overexpression of Notch in neural stem cells induces tumorigenesis, while in intestinal stem cell lineages, increased Notch is required for differentiation (Ohlstein and Spradling 2006; Wang et al. 2006; Ohlstein and Spradling 2007; Bowman et al. 2008).

In the Drosophila testis, Notch signaling was shown to be required for germ cell survival: Delta produced in the germline and Notch in the somatic cyst cells are both required to prevent cell death of the germline (Ng et al. 2019). In light of our results showing that increased Notch signaling in *miR-317* mutants is a prerequisite for tumor formation, it is possible that the levels of Notch components or the strength of Notch signaling must be kept within a particular range to prevent either cell death or overproliferation. Another possibility is that cysts at different developmental stages have variable competence to undergo Notch-mediated germline overproliferation. It has been reported that Notch signaling is not active in the youngest cyst cells near the apex, rather only in more differentiated, slightly more basal cells (Ng et al. 2019). Thus, it is possible that increased Notch signaling strength in earlier cyst cells in the spermatogonial region have the propensity non-autonomously to cause overproliferation of their associated germline cells. Future studies will be required to assess the competence of different stages of cysts to undergo tumorigenesis.

### Regulation of miRNA expression by differential polyadenylation

As described above, several lines of evidence allowed us to conclude that *Peony* and *miR-317* comprise a polycistronic unit and mutually affect one another’s expression levels. Interestingly, our results show that increased expression of *Peony* also results in elevated *miR-317* levels. We note the presence of a predicted TSS at the 5' end of *Peony*, while no additional TSS exists before *miR-317*. Also, three tandem PASs exist between *Peony* and *miR-317*, while none are predicted downstream of *miR-317*. The simplest explanation for these observations is that transcriptional read-through of the proximal PAS is required for expression of the miRNA, and the choice of PAS determines whether *Peony* alone is expressed, or *miR-317* as well. Indeed, in the event that the PAS is bypassed and *miR-317* is transcribed, pre-miRNA processing and cleavage would be predicted to de-stabilize the 5' portion of the transcript that includes *Peony*. Therefore, expression of the two genes could in principle be mutually exclusive: proximal polyadenylation stabilizes the *Peony* transcript but prevents expression of *miR-317*, while transcriptional read-through allows miRNA processing but causes degradation of *Peony*.

An important and exciting question is whether other miRNA genes are regulated in a way similar to the *Peony-miR-317* locus. We looked carefully at the genomic loci of all known Drosophila miRNA genes, of which there are over 260 (Supplemental Table 5; Supplemental Figure 4A). The most common genomic context for miRNA genes is presence in the intron of a coding gene (118/264 miRNA genes, 44.7%). Many others are located in exons or in intergenic space, while an appreciable minority (46/264 genes, 17.4%) reside closely upstream or downstream of adjacent genes in a way similar to the close proximity of *Peony* and *miR-317* (Supplemental Figure 4B). This 17.4% of miRNA genes, which we term “putatively co-transcribed,” represents an intriguing avenue for further investigation to determine if they are indeed co-expressed with adjacent genes and whether alternative polyadenylation may play a role in their differential expression.

### Possible implication of *Peony* in phenotypes attributed to *miR-317*

In light of our finding that the functional lncRNA *Peony* is adjacent to and co-transcribed with *miR-317*, phenotypes that would ordinarily have been ascribed to the miRNA should be scrutinized carefully for involvement of *Peony*. For instance, several studies reported phenotypes that they attributed to loss of *miR-317*, when the allele used was a P{GT1} transposon (BG01900) inserted in *Peony*, 1730 bp 5′ of *miR-317* (Yamamoto et al. 2008; Edwards et al. 2009; Fricke et al. 2014). Moreover, care should be given in interpretation of phenotypes caused by the *miR-317[ko]* allele since, as demonstrated here, this allele results in a strong upregulation of *Peony*, which could produce confounding results. Potentially helpful in this respect is the new *Df-miR-317* allele generated for this work, which removes both *miR-317* and *Peony*, allowing the unambiguous attribution of phenotypes to the miRNA.

### The cryptic yet crucial functions of lncRNAs

It has recently become appreciated that eukaryotic genomes undergo pervasive transcription, in stark contrast to the historical view that the transcriptome consists mainly of protein-coding transcripts and other RNAs involved primarily in translation: tRNAs, rRNAs, and a few others. This widespread transcription results in a vast number of non-coding transcripts, the functions of most of which are still unknown (Jensen et al. 2013). MiRNAs comprise one class of non-coding RNAs (ncRNAs), but while the canonical function of miRNAs is well-known, the same cannot be said for the lncRNAs. Although many thousands of these transcripts exist in animal cells, their functions remain mostly obscure. It is a subject of much debate whether many lncRNAs are the result of transcriptional noise with no bona fide functions within the cell. Counter to this proposition has been the elucidation of important functions for an ever-increasing number of lncRNA genes as well as clear incidences of evolutionary conservation, which would be unexpected for transcripts with no importance (Guttman et al. 2009; Chen 2016; Li and Liu 2019). Indeed, the study of lncRNAs’ molecular and cellular functions and the determination of their roles in living cells remains an important and tantalizing problem in modern biology. Our finding that *Peony* is co-transcribed with an important tumor-suppressive miRNA and that the lncRNA itself also affects muscle integrity and testis morphology adds an important element to the growing body of evidence showing the important roles that lncRNAs play in many different contexts.

## Materials and methods

### Fly Stocks and Genetics

Flies were maintained at 25°C at constant humidity and a 12h-12h light-dark cycle. They were kept on standard cornmeal-yeast-agar fly food. All genotypes used are listed in Key Resources Table.

The following stocks were provided by the Bloomington Drosophila Stock Center (BDSC): *w[1118]* (BDSC 6326); *OR-R* (BDSC 5); *C587-Gal4* (BDSC 67747); *P{UAS-3xFLAG.dCas9.VPR}attP40* (BDSC 66561); *P{w[+mC]=EP} EP3412* (BDSC 17125); *P{w[+mC]=EP}Ada2b[G3857]* (BDSC 31778); *P{y[+t7.7] v[+t1.8]=TOE.GS02678}* (BDSC 79975); w[*]; P{w[+mW.hs]=GawB}bab1[Pgal4-2]/TM6B, Tb[1] (BDSC 6803); *P{w[+mC] EPgy2}CG18265[EY11347]* (BDSC 20298); *P{y[+t7.7] v[+t1.8]=TOE.GS03029}* (BDSC 80311); *P{w[+mGS]=GSV1}EP-820[EP-820]* (BDSC 43450); *P{y[+mDint2] w[+mC]=EPgy2}EY14693* (BDSC 21439); *P{y[+t7.7] v[+t1.8]=TOE.GS03028}* (BDSC 80310); *Mi{MIC}MI02131* (BDSC 37146); *P{w[+mW.hs]=GawB}elav[C155]* (BDSC 458); w*; P{UAS-mam.A}2 (BDSC 27743); *mam[8]/CyO* (BDSC 1596); *miR-317[ko]* (BDSC 58296); *P{w[+mW.hs]=GawB}MJ12a* (BDSC 6991); w[1118]; P{w[+m*]=NRE-EGFP.S}5A (BDSC 30727); *P{y[+t7.7] v[+t1.8]=TOE.GS01580}* (BDSC 85759); *P{w[+mC]=UAS-rn.SP}1* (BDSC 7403); *RpII215[c4]* (BDSC 3663); y1 w*; P{tubP-GAL4}LL7/TM3, Sb1 Ser1 (BDSC 5138); w[*]; P{w[+mC]=tubP-GAL80[ts]}10 (BDSC 7108); and w[*]; P{w[+mC]=tubP-GAL80[ts]}2 (BDSC 7017).

The following stocks were provided by the Kyoto Drosophila Stock Center at the Department of Drosophila Genomics and Genetic Resources (DGGR), Kyoto Institute of Technology: *P{GSV6}GS11364* (DGGR 203275); *P{GSV6}GS14279* (DGGR 205750); *P{GSV6}GS10801* (DGGR 202839); y* w*; P{w+mW.hs=GawB}NP1624 (DGGR 104055); and *P{w+mC=vas-GAL4.2.6}2* (DGGR 109996).

The following stocks were procured from the Exelixis collection at Harvard Medical School: *PBac{WH}f04665, P{XP}d02752, PBac{WH}f01098, P{XP}d04430*, and *P{XP}d09170*.

Other stocks used were: *UAS-Peony* (this study); *Df-miR-317* (this study); *P{w+mC=bam-GFP.−799/+133}3LR* (Chen and McKearin 2003); *P[UASp-Delta-2]* (Ward et al. 2006); w*; P{GAL4-nos.NGT}40; P{GAL4::VP16-nos.UTR}CG6325MVD1 (gift from Hannele Ruohola-Baker); *Mef2-Gal4* (gift from Gerd Vorbrüggen); and *Tub-Gal4* (chromosome 2; gift from Stefan Jakobs). Alleles used to express activated forms of Notch were *UAS-N[cdc10]*, which expresses an intracellular fragment of Notch (Brennan et al. 1999) and *UAS-N[B33c-3]*, which results in the expression of a constitutively active form of Notch lacking most of the extracellular domain (Larkin et al. 1996).

### Immunofluorescence and confocal microscopy

Adult flies were dissected with Dumont #5 forceps (Fine Science Tools) in cold phosphate-buffered saline (PBS). Testes were fixed in 4% formaldehyde (Polysciences 18814-20) in PBT (PBS + 0.2% Triton-X 100 (Sigma 108603)) for 20 minutes, then rinsed in PBT multiple times for a total of at least 30 minutes. Then, testes were blocked in PBTB solution (PBT + 5% normal goat serum (Abcam ab7481) and 0.1% bovine serum albumin (Applichem A1391) for at least 30 minutes. Then, testes were incubated in a solution of primary antibodies diluted in PBTB overnight at 4°C. After primary antibody incubation, testes were rinsed multiple times for at least 30 minutes, blocked in PBTB for at least 30 minutes, and incubated in a solution of secondary antibodies diluted in PBTB, either at room temperature for 2.5 hours or at 4C overnight. Then, testes were rinsed multiple times in PBT, incubated in a 1 μg/mL solution of DAPI in PBT for 10 minutes, and rinsed three more times with PBT. Finally, the buffer was removed and testes were stored in Vectashield (Vector Labs) until being mounted on slides and imaged.

The following mouse monoclonal antibodies were procured from the Developmental Studies Hybridoma Bank and used at the dilutions shown: anti-Adducin (1:20), anti-LaminC (1:20), anti-Dlg (1:20), anti-Eyes absent (1:100), and anti-β-Galactosidase (1:200). Other antibodies used were: chicken anti-GFP (1:5000, Abcam); rabbit anti-mCherry (1:200, Abcam); rabbit anti-Phosphohistone H3 (1:10000, Upstate Biotechnology); guinea pig anti-Traffic jam (1:5000, a gift from Dorothea Godt); guinea pig anti-β3-Tubulin (1:1500, a gift from Renate Renkawitz-Pohl); and rabbit anti-Vasa (1:5000, a gift from Herbert Jäckle). Tissues were also counterstained using the DNA stain DAPI (VWR) to mark nuclei and Cy3-labeled phalloidin (ThermoFisher) to mark actin filaments.

Imaging was performed with a Zeiss LSM 700 confocal microscope using a 10x air objective, a 25x oil objective, or a 40x oil objective. Images were processed using Fiji software (Schindelin et al. 2012) and assembled in Adobe Illustrator (Adobe, Inc.).

### miRNA target prediction

The following databases were used to search for predicted targets of *miR-317*: TargetScanFly (http://www.targetscan.org/fly_12/), microRNA.org (http://www.microrna.org), and MinoTar (https://www.flyrnai.org/cgi-bin/DRSC_MinoTar.pl). 975 targets were predicted by microRNA.org, 102 by TargetScanFly, and 31 by MinoTar; 76 targets were predicted by multiple databases for a total of 1030 predicted targets. These databases consider transcripts of coding genes as potential miRNA targets, so to identify miR-317 binding sites in lncRNAs, we performed a manual curation. The predominant miR-317 species is miR-317-3p, mature sequence UGAACACAGUGCUAAAUGAAAGA. We used the seed sequence (positions 2-7, GAACAC) as well as one base on either side of the seed to define potential targets. According to the TargetScanFly custom, transcripts were considered potential targets if they possessed the following: 8mer site UGUGUUCA; 7mer-m8 site UGUGUUC; 7mer-A1 site GUGUUCA; offset 6mer site UGUGUU; or 6mer site GUGUUC. We downloaded the sequences of all lncRNAs that were dysregulated in testis RNA-seq and searched for these sequences. Of the 192 lncRNAs dysregulated in testes, we found that 56 possess potential miR-317 binding sites (Supplemental Table 1).

### qRT-PCR

Drosophila tissues were dissected in cold PBS and transferred to microcentrifuge tubes. Total RNA was prepared using a standard Trizol extraction followed by an isopropanol precipitation. RNA was quantified using a Nano-drop spectrophotometer (ThermoFisher) and treated with DNaseI. For expression levels of mRNA, lncRNA, or pre-miRNA, SYBR Green-based qPCR was performed. For measuring mature miRNA levels, a TaqMan-based assay was performed. Reactions were run in triplicate. Either α-Tubulin 84B (SYBR Green qPCR) or 2S rRNA (TaqMan qPCR) was used as an endogenous control. Expression fold change (FC) was determined using the expression FC = 2^−ΔΔCt^.

### Generation of *UAS-Peony*

The pUASt-attB vector was linearized with XhoI. The *Peony* cDNA was generated from RNA taken from *w[1118]* flies and PCR amplified using the following primers (lowercase bases indicate overlap to the vector; uppercase bases correspond to genomic sequence): UAS-Peony_F aacagatctgcggccgcggcGAATTCTGAACACAGCAAAAAGTACTGCATATAATAC; UAS-Peony_R2 aagatcctctagaggtacccGCTCAGCAGAGACACAAACCG. Gibson assembly was used to assemble insert and vector. Transgenesis was performed by GenetiVision; the transgene was site-specifically integrated at the VK1 attP site on chromosome 2R.

### RNA-sequencing

RNA was extracted from ovaries and testes from *w[1118]* and *miR-317[ko]* flies. Testes were separated from accessory glands and other components of the male reproductive system, and mature eggs were removed from ovaries prior to RNA extraction. All samples were in triplicate. Total RNA was treated with DNaseI and submitted to the Transcriptome and Genome Analysis Laboratory (TAL) at the Georg-August-University, Göttingen, Germany. RNA quality was assessed by TAL prior to library preparation and sequencing. Sequencing was performed on an Illumina HiSeq4000 system, generating 50-bp reads and approximately 50 million reads per sample. Alignment and differential expression analysis was performed by TAL, resulting in the identification of 2944 genes in ovaries and 1844 genes in testes that were dysregulated in *miR-317[ko]* (Supplemental Tables 1 and 2).

## Figures and Tables

**Table 1:** Predicted *miR-317* targets upregulated in testes in *miR-317[ko]* vs control; p<0.05 and Log_2_FC>0.5

Gene ID	Name	Protein Description	Log_2_ Fold Change	Adjusted p-value
FBgn0037427	*Osi17*	Osiris 17 ; integral component of plasma membrane	1.29	4.12E-09
FBgn0037419	*Osi12*	Osiris 12 ; integral component of plasma membrane	1.22	2.53E-08
FBgn0039178	*CG6356*	CG6356 ; transmembrane transport	1.05	2.63E-11
FBgn0066114	*GlcAT-I*	GlcAT-I ; Golgi membrane	1.02	9.05E-11
FBgn0036661	*CG9705*	Cold shock domain-containing protein CG9705 ; regulation of transcription, DNA-templated	0.81	1.03E-21
FBgn0037845	*CG14694*	IP11787p ; transmembrane transporter activity	0.79	1.62E-04
FBgn0267337	*rn*	Rotund ; transcription factor activity	0.69	3.58E-11
FBgn0031001	*CG7884*	CG7884, isoform B ; no biological data available	0.68	1.44E-03
FBgn0034789	*PIP5K59B*	PIP5K59B ; Phosphatidylinositol phosphate kinase activity	0.67	3.30E-03
FBgn0036725	*CG18265*	GH10077p ; nucleic acid binding	0.67	2.46E-03
FBgn0037555	*Ada2b*	Transcriptional Adaptor 2b ; regulation of transcription, DNA-templated	0.61	6.22E-07
FBgn0039064	*CG4467*	CG4467, isoform B ; proteolysis	0.58	2.93E-02
FBgn0037837	*CG14693*	Uncharacterized protein, isoform E	0.56	3.38E-08
FBgn0031275	*GABA-B-R3*	Metabotropic GABA-B receptor subtype 3 ; G-protein coupled receptor signaling pathway	0.54	1.98E-08
FBgn0033476	*oys*	Oysgedart ; lysophospholipid acyltransferase activity	0.53	1.46E-09

**Table 2:** Testing for GLT formation caused by overexpression (OE) of candidate genes

Gene	OE line	BDSC#	Type of OE line	Notes on OE line	Germline OE	Somatic OE	FLP-out OE
Predicted *miR-317* target genes upregulated in testes							
*rn*	*P{w[+mC]=UAS-rn.SP}1*	7403	in vitro-constructed UAS-gene fusion	*UAS-rn* (ii)	no tumors	some tumors	no tumors
*CG18265*	*P{w[+mC] EPgy2}CG18265 [EY11347]*	20298	UAS-containing transposon insertion	inserted in 5'UTR intron	no tumors	some tumors	n.d.*
*CG18265*	*P{y[+t7.7] v[+t1.8]=TOE.G S03029}*	80311	CRISPR-OE line	CG18265 OE gRNA	n.d.	some tumors	n.d.
*Ada2b*	*P{w[+mC]=EP}E P3412*	17125	UAS-containing transposon insertion	upstream of *Ada2b*	no tumors	no tumors	n.d.
*Ada2b*	*P{w[+mC]=EP}A da2b[G3857]*	31778	UAS-containing transposon insertion	inserted in 5'UTR	no tumors	no tumors	n.d.
*Ada2b*	*P{y[+t7.7] v[+t1.8]=TOE.G S02678}*	79975	CRISPR-OE line	*Ada2b* OE gRNA	n.d.	no tumors	n.d.
*CG4467*	*P{w[+mGS]=GS V1}EP-820[EP-820]*	43450	UAS-containing transposon insertion	inserted upstream of *CG4467*	no tumors	no tumors	n.d.
*CG6356*	*P{y[+mDint2] w[+mC]=EPgy2} EY14693*	21439	UAS-containing transposon insertion	*CG6356* OE	no tumors	no tumors	n.d.
*CG9705*	*P{y[+t7.7] v[+t1.8]=TOE.G S03028}*	80310	CRISPR-OE line	*CG9705* OE gRNA	n.d.	no tumors	n.d.
*PIP5K59B*	*P{y[+t7.7] v[+t1.8]=TOE.G S01580}*	85759	CRISPR-OE line	*PIP5K59B* OE gRNA	n.d.	no tumors	n.d.
Notch-related genes							
*Delta*	*UASp-Delta*	NA	in vitro-constructed UAS-gene fusion	OE full-length Dl	no tumors	n.d.	n.d.
*Notch*	*UAS-N[cdc10]*	NA	in vitro-constructed UAS-gene fusion	OE of NICD	lethal	mostly lethal; no tumors	no tumors
*Notch*	*UAS-N[B33c-3]*	NA	in vitro-constructed UAS-gene fusion	OE constitutively active N	lethal	mostly lethal; no tumors	mostly lethal; no tumors
*mam*	w*; *P{UAS-mam.A}2*	27743	in vitro-constructed UAS-gene fusion	n.d.	n.d.	some tumors	some tumors
*Peony* OE							
*Peony*	*UAS-Peony*	NA	in vitro-constructed UAS-gene fusion	OE full-length *Peony* cDNA	no tumors	no tumors	no tumors

* n.d., experiment not done

**Table:** Key Resources Table

Reagent type	Source	Identifier	Gene
Drosophila stock	BDSC #5	*OR-R*	-
Drosophila stock	BDSC #67747	*C587-Gal4*	-
Drosophila stock	BDSC #66561	*P{UAS-3xFLAG.dCas9.VPR}attP40*	-
Drosophila stock	BDSC #17125	*P{w[+mC]=EP}EP3412*	*Ada2b*
Drosophila stock	BDSC #31778	*P{w[+mC]=EP}Ada2b[G3857]*	*Ada2b*
Drosophila stock	BDSC #79975	*P{y[+t7.7] v[+t1.8]=TOE.GS02678}*	*Ada2b*
Drosophila stock	BDSC #6803	w[*]; P{w[+mW.hs]=GawB}bab1[Pgal4-2]/TM6B, Tb[1]	*bab1-Gal4*
Drosophila stock	BDSC #20298	*P{w[+mC] EPgy2}CG18265[EY11347]*	*CG18265*
Drosophila stock	BDSC #80311	*P{y[+t7.7] v[+t1.8]=TOE.GS03029}*	*CG18265*
Drosophila stock	BDSC #43450	*P{w[+mGS]=GSV1}EP-820[EP-820]*	*CG4467*
Drosophila stock	BDSC #21439	*P{y[+mDint2] w[+mC]=EPgy2}EY14693*	*CG6356*
Drosophila stock	BDSC #80310	*P{y[+t7.7] v[+t1.8]=TOE.GS03028}*	*CG9705*
Drosophila stock	BDSC #37146	*Mi{MIC}MI02131*	*Peony*
Drosophila stock	BDSC #458	*P{w[+mW.hs]=GawB}elav[C155]*	*elav-Gal4*
Drosophila stock	BDSC #27743	w*; P{UAS-mam.A}2	*mastermind*
Drosophila stock	BDSC #1596	*mam[8]/CyO*	*mastermind*
Drosophila stock	BDSC #58296	*miR-317[ko]*	*miR-317*
Drosophila stock	BDSC #6991	*P{w[+mW.hs]=GawB}MJ12a*	*MJ12a-Gal4*
Drosophila stock	BDSC #30727	w[1118]; P{w[+m*]=NRE-EGFP.S}5A	*NRE-GFP*
Drosophila stock	BDSC #85759	*P{y[+t7.7] v[+t1.8]=TOE.GS01580}*	*PIP5K59B*
Drosophila stock	BDSC #7403	*P{w[+mC]=UAS-rn.SP}1*	*rotund*
Drosophila stock	BDSC #3663	*RpII215[c4]*	*RpII215*
Drosophila stock	BDSC #5138	y1 w*; P{tubP-GAL4}LL7/TM3, Sb1 Ser1	*Tub-Gal4 (III)*
Drosophila stock	BDSC #7108	w[*]; P{w[+mC]=tubP-GAL80[TS]}10	*Tub-Gal80[TS] (II)*
Drosophila stock	BDSC #7017	w[*]; P{w[+mC]=tubP-GAL80[TS]}2	*Tub-Gal80[TS] (III)*
Drosophila stock	BDSC #6326	*w[1118]*	*white*
Drosophila stock	DGGR #203275	*P{GSV6}GS11364*	*Peony*
Drosophila stock	DGGR #205750	*P{GSV6}GS14279*	*Peony*
Drosophila stock	DGGR #202839	*P{GSV6}GS10801*	*Peony*
Drosophila stock	DGGR #104055	y* w*; P{w+mW.hs=GawB}NP1624	*tj-Gal4*
Drosophila stock	DGGR #109996	*P{w^+mC^=vas-GAL4.2.6}2*	*vasa-Gal4*
Drosophila stock	Exelixis #f04665	*PBac{WH}f04665*	*Peony*
Drosophila stock	Exelixis #d02752	*P{XP}d02752*	*Peony*
Drosophila stock	Exelixis #f01098	*PBac{WH}f01098*	*Peony*
Drosophila stock	Exelixis #d04430	*P{XP}d04430*	*Peony*
Drosophila stock	Exelixis #d09170	*P{XP}d09170*	*Peony*
Drosophila stock	this study	*UAS-Peony*	*Peony*
Drosophila stock	this study	*Df-miR-317*	*miR-317*
Drosophila stock	(Brennan et al, 1999)	*UAS-N[cdc10]*	*Notch*
Drosophila stock	(Chen & McKearin, 2003)	*P{w+mC=bam-GFP.−799/+133}3LR*	*bam*
Drosophila stock	(Larkin et al, 1996)	*UAS-N[B33c-3]*	*Notch*
Drosophila stock	(Ward, et al. 2006)	*P[UASp-Delta-2]*	*Delta*
Drosophila stock	Gift from Hannele Ruohola-Baker	w*; P{GAL4-nos.NGT}40; P{GAL4::VP16-nos.UTR}CG6325MVD1	*nos-Gal4*
Drosophila stock	Gift from Gerd Vorbrüggen	*Mef2-Gal4*	*Mef2-Gal4*
Drosophila stock	Gift from Stefan Jakobs	*Tub-Gal4*	*Tub-Gal4 (II)*
Antibody	DSHB	Mouse anti-Adducin 1:20	NA
Antibody	DSHB	Mouse anti-Eyes absent 1:100	NA
Antibody	DSHB	Mouse anti-β-Galactosidase 1:200	NA
Antibody	DSHB	Mouse anti-LaminC 1:20	NA
Antibody	Abcam	Chicken anti-GFP 1:5000	NA
Antibody	Abcam	Rabbit anti-mCherry 1:200	NA
Antibody	Upstate Biotechnology	Rabbit anti-Phosphohistone H3 1:10000	NA
Antibody	Gift from Dorothea Godt	Guinea pig anti-Traffic jam 1:5000	NA
Antibody	Gift from Renate Renkawitz-Pohl	Guinea pig anti-β3-Tubulin 1:1500	NA
Antibody	Gift from Herbert Jäckie	Rabbit anti-Vasa 1:5000	NA
Stain	VWR	DAPI	NA
Stain	Thermofisher	Cy3-labeled Phalloidin	NA

DGGR = Department of Drosophila Genomics and Genetic Resources, Kyoto Institute of Technology

BDSC = Bloomington Drosophila Stock Center

DSHB = Developmental Studies Hybridoma Bank
